# Recent Advances in Pretreatment Methods and Detection Techniques for Veterinary Drug Residues in Animal-Derived Foods

**DOI:** 10.3390/metabo15040233

**Published:** 2025-03-28

**Authors:** Qing Dai, Shusheng Tang, Chongshan Dai

**Affiliations:** 1 National Key Laboratory of Veterinary Public Health and Safety, College of Veterinary Medicine, China Agricultural University, Beijing 100193, China; 2 Technology Innovation Center for Food Safety Surveillance and Detection (Hainan), Sanya Institute of China Agricultural University, Sanya 572025, China; 3 China Institute of Veterinary Drug Control, Beijing 100081, China

**Keywords:** animal derived foods, veterinary drug residues, pretreatment methods, detection techniques

## Abstract

Veterinary drugs are extensively employed in livestock, poultry, and aquaculture, playing a crucial role in preventing and treating animal diseases, facilitating growth, and enhancing feed conversion rates. Nevertheless, veterinary drug residues in animal-derived foods pose a direct or potential threat to human life and health. Precise detection of these residues in animal-derived foods to ensure their safety has become an important mission. In this review, we sum up the current progress of applied pretreatment methods and detection techniques for veterinary drug residues in animal-derived foods. At present, sample pretreatment methods mainly consist of the following: liquid–liquid extraction; solid-phase extraction; immunoaffinity chromatography; Quick, Easy, Cheap, Effective, Rugged, and Safe (QuEChERS) method; and molecular imprinting technology. Detection techniques mainly involve chromatographic techniques, immunoassay techniques, fluorescence polarization immunoassay, and surface-enhanced Raman scattering. We also discussed the advantages and limitations of these technologies. Moreover, we point out the development direction and tendency of detection techniques in the future, providing references for the detection of veterinary drug residues in animal-derived foods.

## 1. Introduction

Veterinary drugs utilized for disease treatment and prevention might linger in animal-derived foods, like milk, eggs, honey, and meat, which could endanger the public’s health [1,2,3]. However, some illegal enterprises, for the sake of swift profit seeking, fail to abide by the medication instructions and use veterinary drugs or even prohibited drugs beyond their scope, dosage, and treatment duration. These actions will exert complex and far-reaching impacts on target animal safety, contact safety, environmental and ecological safety, animal food safety, and public health safety [4,5]. Veterinary drug residues refer to the residual substances associated with drugs that accumulate in an animal’s body post-treatment with medication, encompassing drug prototypes and their metabolites [6,7]. Veterinary drug residues do not remain at the food processing stage; instead, they accumulate along the food chain. They not only contaminate the environment but also inflict substantial harm to the human body when amassed in a certain quantity over a long period [8,9]. Consequently, strict supervision of veterinary drug residues is essential to control the safety and quality of animal-derived foods and guarantee consumer safety [10,11].

Recent advancements in the detection of veterinary drug residues have predominantly focused on edible animals and their by-products, including organs, eggs, bee products, and aquatic species such as fish and shrimp [12,13,14,15]. The complexity of animal-derived food matrices, characterized by numerous interfering substances, necessitates the development of both efficient pretreatment methodologies and highly sensitive and specific detection technologies [16,17,18]. In the global context, various nations have adopted divergent approaches to veterinary drug residue detection, influenced by their respective food safety regulations and technological infrastructures. In China, liquid chromatography–mass spectrometry (LC-MS) is recognized as the “golden standard” due to its superior sensitivity and precision. Additionally, China is actively advancing and implementing rapid detection technologies such as Gold Immunochromatographic Assay (GICA) and Enzyme-Linked Immunosorbent Assay (ELISA) [19]. The trajectory of veterinary drug residue detection technology in China is increasingly oriented towards high-throughput and high-sensitivity methodologies. The United States extensively utilizes LC-MS for veterinary drug residue detection, leveraging its remarkable sensitivity and accuracy [20,21]. Concurrently, the U.S. is actively researching and applying biosensor and biochar technologies, which are gaining significant attention for their rapid and high-throughput capabilities [22]. European countries also place substantial emphasis on the application of LC-MS in veterinary drug residue detection [23]. There has been extensive research and implementation of pretreatment methods such as Quick, Easy, Cheap, Effective, Rugged, and Safe (QuEChERS) and solid-phase extraction (SPE) [24]. Microbiological testing methods are widely employed in Europe, particularly for the rapid screening of antibiotic residues [25]. Given the stringent food safety regulations in Europe, the region boasts advanced and diverse veterinary drug residue detection technologies.

For this review, the most important articles published from 1 January 2000 to 1 January 2025 relating to pretreatment methods and detection techniques for veterinary drug residues in animal-derived foods were selected from the Scopus, PubMed, and Web of Science databases. The keyword combinations used for the literature search are as follows: ‘veterinary drug residues and pretreatment methods’, ‘veterinary drug residues and animal-derived foods’, or ‘drug residues and animal-derived foods’. For each pretreatment method or analysis method, some representative publications were further selected.

This paper provides a comprehensive review of recent pretreatment methods and detection techniques for animal-derived foods, intending to contribute to the future development of veterinary drug residue detection technologies. By examining the current landscape and identifying key trends, we aim to offer valuable insights and recommendations for enhancing the efficacy and applicability of these critical methodologies.

## 2. Pretreatment Methods

Animal-derived foods possess complex matrices, high concentrations of interfering substances, and low residue levels, which render their separation and purification arduous [26]. They fall into the category of detection technologies for trace components in complex matrix samples. In a comprehensive detection experiment, pretreatment is the most time-consuming and crucial task, directly affecting the precision and accuracy of the experiment [27]. Pretreatment methods for veterinary drug residues mainly consist of extraction, separation, purification, concentration, and enrichment. Extraction and purification are fundamental steps since they can remove matrix interference and safeguard instruments [28,29]. Currently, establishing a rapid and efficient sample pretreatment method has become the development trend and challenge for veterinary drug residue analysis techniques.

### 2.1. Liquid–Liquid Extraction (LLE) Technology

LLE is a separation technique based on the principle of differential distribution coefficients of solutes in two immiscible solvent systems [30]. When a sample extract containing the target veterinary drug is brought into contact with a selected organic solvent, the drug molecules distribute themselves between the organic and aqueous phases according to their solubility, which is determined by their chemical properties [30]. This distribution, governed by the partition coefficient, enables the initial separation and migration of the veterinary drug toward the organic phase, where it is more soluble [30]. LLE is widely recognized for its operational simplicity and effectiveness in extracting target analytes from complex matrices. However, it consumes a significant volume of organic solvents, which not only increases operational costs but also raises safety and environmental concerns due to the flammable, explosive, and toxic nature of many solvents [31,32]. Additionally, LLE is rarely used alone for the pretreatment of animal-derived foods with complex matrices, and often requires combining it with other pretreatment methods to achieve optimal results [33].

The advantages of LLE include its excellent selectivity, which leverages the chemical properties of veterinary drugs to choose compatible organic solvents, such as chloroform for lipophilic drugs. This enables precise separation from complex matrices like animal tissues and milk, effectively reducing interference from hydrophilic impurities [30]. The method is also operationally simple, requiring minimal equipment—common separatory funnels are sufficient—and involves straightforward steps such as manual shaking and phase separation, making it accessible for routine use [34,35]. Furthermore, LLE is cost-effective, utilizing low-cost organic solvents like acetonitrile and ethyl acetate, combined with simple equipment, resulting in a low initial investment for detection [35].

Despite its advantages, LLE has notable limitations. The high consumption of organic solvents not only increases costs but also poses safety risks and environmental concerns due to their hazardous nature [36]. Emulsification is another common issue, particularly in samples containing proteins or surfactants, where the organic and aqueous phases may form stable emulsions that resist separation, necessitating additional demulsification steps and complicating the process [35]. Additionally, the extraction efficiency of LLE can be limited, especially for drugs with similar partition coefficients. A single extraction may be insufficient, requiring multiple extraction steps that are time-consuming and labor-intensive, thereby reducing overall efficiency [37].

Several studies have demonstrated the application of LLE in veterinary drug residue analysis. For instance, Sun et al. employed Na_2_EDTA-Mcllvaine buffer and acetonitrile as extraction solvents, along with anhydrous MgSO_4_ and NaCl as extraction salts, in a salt-precipitation-assisted LLE method combined with high-performance liquid chromatography–tandem mass spectrometry (HPLC-MS/MS). This approach enabled the detection of 22 veterinary drug residues in aquatic products, with detection limits ranging from 0.5 to 1.0 μg/kg and accuracy between 71.4% and 120% [38]. Similarly, Wang et al. utilized methanol and acetonitrile as extraction solvents, performing static extraction three times. The preliminarily purified extract was further refined using magnetic materials and analyzed by ultra-performance liquid chromatography–tandem mass spectrometry (UPLC-MS/MS). Under optimized conditions, this method identified 33 types of antibiotics and 37 types of pesticides [39]. In summary, while LLE offers significant advantages in terms of selectivity, simplicity, and cost-effectiveness, its limitations, including high solvent consumption, emulsification risks, and limited extraction efficiency, necessitate careful consideration and often require complementary techniques to achieve reliable and efficient results in veterinary drug residue analysis [40].

### 2.2. SPE

SPE is a widely used sample preparation technique based on the principles of adsorption and desorption. It is also a technique designed for rapid and selective sample preparation and purification prior to chromatographic analysis [41]. When a sample extract passes through an extraction column packed with a solid-phase adsorbent, veterinary drugs are selectively adsorbed onto the active sites of the adsorbent surface due to their specific chemical structures and properties. The adsorption strength varies depending on the polarity and functional groups of the veterinary drugs, allowing for selective retention of target analytes [42]. During this process, impurities with weaker adsorption affinities are washed away with the eluent, achieving preliminary purification. Subsequently, an appropriate elution solvent is used to desorb the target veterinary drugs from the adsorbent, and the eluate is collected for subsequent analysis [39,40]. Since its development, SPE has been extensively applied in various fields due to its simplicity, high efficiency, and rapidity. Compared to traditional LLE, SPE is faster, more solvent efficient, and exhibits better reproducibility, making it the preferred method for sample pretreatment [43,44].

SPE offers several notable advantages. First, it provides effective enrichment of trace amounts of veterinary drugs. For example, in water samples, SPE can concentrate extremely low drug concentrations, significantly enhancing detection sensitivity and enabling compliance with stringent regulatory limits [45]. Second, it exhibits high selectivity. By choosing appropriate adsorbents, target analytes can be precisely adsorbed, effectively excluding a large number of interfering impurities and achieving excellent purification [46]. Third, SPE consumes significantly less solvent compared to LLE, reducing operational costs, environmental pollution, and safety risks [39,40]. Several studies have demonstrated the effectiveness of SPE in veterinary drug residue analysis. For instance, Zhu et al. employed an ethylene diamine tetraacetic acid (pH 7)-acetonitrile (30:70) solvent system combined with a one-step SPE cleanup method. Using UPLC-MS/MS, they simultaneously determined multiple veterinary drugs with excellent sensitivity, achieving limits of quantification ranging from 0.2 μg/kg to 3.0 μg/kg [47]. Similarly, Mehl et al. developed a high-throughput planar SPE cleaning method to screen 81 veterinary drugs from six different groups (glucocorticoids, dulcimers, antiparasitic drugs, antibacterial drugs, non-steroidal anti-inflammatory drugs, and antibiotics) in four matrices (honey, pig muscle, milk, and eggs). This method consumed 13 times less solvent, was more environmentally friendly, and was five times faster than conventional methods [48].

However, SPE also has some limitations. The high cost of specialized, high-performance adsorbents can increase the initial investment for detection. Additionally, the operational procedure is relatively complex, involving multiple steps such as column packing, conditioning, sample loading, washing, and elution [49]. Each step requires strict control of conditions to avoid affecting recovery rates, demanding a high level of technical proficiency from operators [50]. Furthermore, SPE is susceptible to matrix effects, particularly in complex sample matrices such as animal tissue extracts. Matrix components may occupy adsorption sites or compete with veterinary drugs for adsorption, reducing extraction efficiency and interfering with the accuracy of detection results. As a result, additional optimization steps are often required to mitigate these effects [43,44].

In summary, SPE is a powerful and versatile sample preparation technique that offers significant advantages in terms of enrichment, selectivity, and solvent efficiency [51]. However, its limitations, including high adsorbent costs, complex operational procedures, and susceptibility to matrix effects, necessitate careful optimization and skilled execution to ensure reliable and accurate results in veterinary drug residue analysis.

### 2.3. Immunoaffinity Chromatography (IAC)

IAC is a highly sophisticated analytical technique that exploits the specific immunological interactions between antigens and antibodies for the selective extraction and purification of target analytes [52,53,54]. This method involves immobilizing monoclonal or polyclonal antibodies specific to veterinary drugs onto a solid-phase matrix to create an immunoaffinity column [55]. When a sample extract containing veterinary drug residues passes through the column, the target drug, acting as an antigen, binds specifically to the immobilized antibodies through precise molecular recognition mechanisms, forming stable antigen–antibody complexes. Concurrently, other components in the sample matrix that lack affinity for the antibodies are eluted with the mobile phase, achieving initial separation and purification. Subsequently, the environmental conditions are altered using an elution buffer to disrupt the antigen–antibody interactions, allowing for the collection of the eluted veterinary drug for precise downstream analysis [52,53,54,56].

IAC offers several notable advantages. First, it exhibits exceptional specificity due to the precise molecular recognition capability of antibodies for their target veterinary drugs [57]. This enables efficient extraction of drugs from complex biological matrices such as animal tissue homogenates, blood, and urine, significantly reducing matrix interference and enhancing detection accuracy. Second, IAC demonstrates high sensitivity, capable of capturing even trace amounts of veterinary drug residues, making it particularly suitable for stringent regulatory requirements in food safety monitoring. Third, this technique is operationally efficient; once the immunoaffinity column is prepared, the loading and elution procedures are straightforward and time-efficient, facilitating high-throughput analysis [52,53,54]. Several studies have demonstrated the effectiveness of IAC in veterinary drug residue analysis. For example, Peng et al. developed an IAC method for the selective purification of 3-methylquinoxaline-2-carboxylic acid from pig muscle and liver, coupled with high-performance liquid chromatography–ultraviolet detection (HPLC-UV). The method achieved detection limits of 1.0–3.0 μg/kg and quantification limits of 4.0–10.0 μg/kg, with an average recovery rate of 80.1–87.7% and a relative standard deviation of less than 8.5% [58]. Similarly, Wang et al. developed an IAC clean-up method combined with HPLC-MS/MS for the simultaneous determination of chloramphenicol, zearalanone, α-zearalanol, β-zearalanol, zearalenone, α-zearalenol, and β-zearalenol in pig muscle. The method demonstrated excellent linearity (r ≥ 0.9990), precision (relative standard deviation < 2.9%), average recovery (74.5–105.0%), and detection limits (0.04–0.10 μg/kg), making it rapid, reliable, sensitive, and highly applicable to real samples [59].

However, IAC also presents certain limitations. The development of specific antibodies, whether it is monoclonal or polyclonal, requires significant technical expertise and financial investment [60]. Additionally, antibody stability is sensitive to environmental factors necessitating strict storage conditions to maintain functionality. Another limitation is the constrained lifespan of immunoaffinity columns. Repeated use leads to decreased antibody activity and reduced binding capacity, requiring frequent column replacements and increasing operational costs [61]. Furthermore, cross-reactivity with structural analogs in sample matrices may interfere with antibody recognition, potentially leading to false positives [57]. Therefore, supplementary analytical methods are often required to validate results and ensure data reliability.

In short, IAC is a powerful tool for veterinary drug residue analysis, offering exceptional specificity, sensitivity, and operational efficiency. However, its application requires careful consideration of its limitations, including the complexity of antibody development, column lifespan constraints, and potential cross-reactivity [62]. By addressing these challenges, IAC can be effectively integrated into analytical workflows to ensure accurate and reliable detection of veterinary drug residues in complex matrices.

### 2.4. QuEChERS

The QuEChERS method has emerged as a highly regarded pretreatment technique for the detection of veterinary drug residues, primarily based on dispersive solid-phase extraction (d-SPE) technology [61]. The process begins with the addition of an organic solvent, such as acetonitrile, to the sample to dissolve and extract target veterinary drugs from complex matrices like animal muscles, organs, and feed [61]. Concurrently, desiccants like anhydrous magnesium sulfate are introduced to facilitate rapid phase separation between the aqueous and organic layers, minimizing moisture interference in subsequent analyses. Following this, a dispersive solid-phase sorbent is added, which selectively adsorbs impurities such as organic acids, pigments, fats, and sugars from the sample extract, while the target veterinary drugs remain in the organic phase [63,64]. This step ensures a high degree of purification, making the method particularly effective for complex sample matrices.

The QuEChERS method offers several notable advantages. First, it is simple and fast, requiring only basic laboratory equipment such as centrifuge tubes and vortex mixers. This allows for the completion of multiple steps in a short timeframe, significantly enhancing pretreatment efficiency. Second, it is cost-effective, as the organic solvents, desiccants, and sorbents used are relatively inexpensive, making the method suitable for large-scale sample analysis. Third, it provides effective purification by efficiently removing various interfering impurities, leading to more accurate and reliable detection results. These characteristics make QuEChERS particularly well-suited for analyzing complex matrices in veterinary drug residue detection. Several studies have demonstrated the effectiveness of the QuEChERS method in veterinary drug residue analysis. For instance, Yang et al. employed an improved QuEChERS method to determine the residues of 192 veterinary drugs. This method involved efficient extraction with 90% acetonitrile, dehydration with sodium sulfate and acetic acid, and subsequent cleanup using a dispersive solid-phase extraction sorbent, primary secondary amine (PSA). The method was suitable for samples with varying fat contents, providing detection limits ranging from 0.5 to 10 μg/kg, high recovery rates (60–120%), and a low relative standard deviation (<20%) [65]. Similarly, Wang et al. established quantitative methods for 146 types of veterinary drug residues in beef and chicken samples. After enzymatic hydrolysis, the samples were treated with an optimized QuEChERS method. The calibration curves exhibited good linearity, with correlation coefficients ranging from 0.9921 to 0.9994. The recovery rates were within 52.1–138.2%, and the relative standard deviation was 0.4–17.7%. The limits of detection and quantification were 0.15–3.03 μg/kg and 0.5–10 μg/kg, respectively [66]. Mu et al. established a rapid method for the simultaneous determination of 14 antiviral drugs and related metabolites, including amantadine, rimantadine, oseltamivir, oseltamivir carboxylate, memantine, abidol, moxifloxacin, acyclovir, ganciclovir, famciclovir, penciclovir, ribavirin, and immunomodulators, in chicken meat using QuEChERS and liquid chromatography–mass spectrometry techniques. The improved QuEChERS method was used, and the determination time of the target compound was less than 11 min, with a recovery rate of 56.2% to 113.4% [67].

However, the QuEChERS method also has certain limitations. The selectivity of the sorbents is not absolute, meaning that for certain structurally unique impurities or veterinary drugs, the desired adsorption or retention may not be achieved, potentially compromising detection accuracy [68]. Additionally, optimization can be challenging, as different types of veterinary drugs and sample matrices require adjustments in the types and quantities of desiccants and sorbents. This process can be time-consuming and labor-intensive, often relying heavily on empirical knowledge. Furthermore, residual matrix components may still interfere with subsequent analyses even after purification, necessitating additional measures such as the use of internal standards to mitigate these effects and ensure the precision of the detection results [58,59].

In short, the QuEChERS method provides a robust, efficient, and cost-effective solution for the pretreatment of samples in veterinary drug residue analysis. Its simplicity, speed, and effective purification make it highly suitable for complex matrices [69]. However, its limitations, including sorbent selectivity issues, optimization challenges, and residual matrix effects, necessitate careful optimization and supplementary techniques to ensure accurate and reliable detection results [70]. By addressing these challenges, the QuEChERS method can be effectively integrated into analytical workflows for veterinary drug residue detection.

### 2.5. Molecular Imprinting Technology (MIT)

MIT has emerged as a cutting-edge strategy for detecting veterinary drug residues, leveraging its unique molecular recognition capabilities and pretreatment efficiency [71]. At its core, MIT relies on the formation of reversible complexes between template molecules (target veterinary drugs) and functional monomers in solution, driven by covalent or non-covalent interactions. Polymerization is subsequently initiated by cross-linkers, producing a rigid three-dimensional polymeric network—termed Molecularly Imprinted Polymers (MIPs)—that mirrors the template’s structural and chemical features. Following template removal via elution, MIPs retain complementary cavities that exhibit high specificity for the target molecules. During analysis, these cavities selectively adsorb veterinary drug residues from complex matrices through spatial and chemical affinity, enabling efficient separation from interfering substances [72,73]. This molecular recognition mechanism positions MIPs as powerful tools for sample pretreatment in residue analysis.

Recent studies highlight MIT’s applicability in veterinary drug monitoring. For instance, Song et al. synthesized a tetracycline-imprinted MIP via precipitation polymerization using methacrylic acid as a functional monomer. Coupled with dispersive SPE and high-performance liquid chromatography–mass spectrometry HPLC-MS, the method enabled the simultaneous detection of seven macrolide antibiotics in pork. At spiked concentrations of 5–100 mg/kg, recoveries ranged from 68.6% to 95.5%, with intra- and inter-day precision (relative standard deviations, RSDs) below 8%. The method achieved detection limits (LODs) of 0.2–0.5 mg/kg and quantification limits (LOQs) of 0.5–2.0 mg/kg, demonstrating both sensitivity and reproducibility [74]. In another study, Tang et al. developed a clenbuterol-imprinted MIP-coated stirrer for β-receptor agonist analysis in animal-derived foods. Integrating stir bar sorptive extraction with HPLC-MS/MS, the method achieved recoveries of 75.8–97.9% in pork and liver samples, with RSDs of 2.6–5.3%. The technique exhibited exceptional sensitivity, with LODs and LOQs as low as 0.05–0.15 μg/kg and 0.10–0.30 μg/kg, respectively [75].

MIT offers three principal advantages in veterinary drug detection. First, MIPs exhibit customized specificity: their tailored cavities enable precise recognition of target analytes even in complex biological matrices (e.g., tissues, blood, and milk), minimizing matrix interference and enhancing sensitivity. Second, MIPs demonstrate robust stability, retaining structural integrity across diverse temperatures, pH ranges, and organic solvents, which permits reuse and reduces operational costs. Third, the technology is highly adaptable: by modulating template molecules, monomers, and cross-linkers, MIPs can be engineered for diverse veterinary drugs, broadening their applicability [76,77]. However, MIT faces notable challenges. The synthesis process remains labor-intensive, requiring meticulous optimization of polymerization conditions and post-treatment steps, which demands specialized expertise and prolongs development timelines. Additionally, while MIPs exhibit high specificity, cross-reactivity with structurally analogous compounds may occur, potentially yielding false positives [78]. Thus, confirmatory techniques (e.g., mass spectrometry) are often indispensable for validation [79].

In short, MIT represents a transformative approach for veterinary drug residue analysis, combining selectivity, stability, and versatility. While its current limitations necessitate complementary methods for verification, ongoing advancements in polymer design and automation hold promise for streamlining MIP synthesis and enhancing reliability in complex matrices [80].

A comprehensive systematic comparison of these pretreatment methods, encompassing LOD, accuracy, advantages, limitations, feasibility, and cost-effectiveness for veterinary drug residue analysis in animal-derived food matrices, is presented in Table 1. For example, LLE demonstrates particular efficacy for high-fat matrices [81], albeit with prolonged processing times (typically 2–4 h) and substantial solvent-related expenses. SPE enables faster sample preparation (30–60 min) through diverse adsorbent selections, yet incurs significant material costs. IAC, while providing exceptional post-purification efficiency, faces challenges in antibody development costs and cross-reactivity risks and cross-reactivity risks for complex matrices. The QuEChERS method provides rapid and cost-efficient analysis for complex matrices, whereas MIT achieves remarkable specificity (85–95% recognition accuracy) despite complex polymer synthesis requirements [82].

## 3. Chromatographic Detection Techniques

Animal-derived food contains relatively low levels of veterinary drug residues and large quantities of interfering substances, which makes it a challenge to detect trace components in complex matrix samples [83,84]. At present, the detection of veterinary drug residues in animal-based foods mainly relies on chromatography–mass spectrometry technology. Commonly used techniques encompass gas chromatography–mass spectrometry (GS-MS), LC-MS, and capillary electrophoresis–mass spectrometry (CE-MS). In brief, the traditional approaches for detecting veterinary drug residues are typically based on laboratory tests using large-scale instrument analysis [85]. Although these methods offer abundant qualitative and quantitative techniques and relatively high detection accuracy, they have drawbacks such as necessitating complex pre-processing methods, requiring professional training for testers, and having relatively expensive equipment, which are not favorable for large-scale and rapid daily risk monitoring. As science and technology progress, convenient and highly sensitive rapid detection methods for veterinary drug residues have gradually become the chief means of daily risk monitoring [86].

### 3.1. GC-MS

GC-MS is a robust analytical technique that integrates the separation capabilities of GC with the detection features of MS [87]. This process begins with the gasification of the sample, where the analyte is introduced into the GC column. The stationary phase in the GC column selectively retains components based on their physicochemical properties, primarily their volatility and interaction with the stationary phase. Following separation in the GC column, the analytes are introduced into the MS, where they undergo ionization, and their molecular ions are detected based on their mass-to-charge ratio (m/z) [88]. This dual functionality of GC-MS enables both separation and identification, making it highly suitable for detecting and quantifying trace volatile organic compounds (VOCs) and other small molecules such as antibiotics in complex matrices, such as food and pharmaceutical samples [89,90]. For instance, Liu et al. developed a novel pre-column derivatization GC-MS/MS method to determine residual decanoate in chicken tissues (muscle, liver, and kidney) [91]. The sample was extracted and purified through liquid–liquid extraction combined with solid-phase extraction and derivatized using acetic anhydride and pyridine. The recovery rate of decanoic acid ester ranged from 77.38% to 89.65%, with intra-day and inter-day relative standard deviations of 1.63–5.74% and 2.27–8.06%, respectively. This method was applied to analyze the tissues of 60 chickens purchased from nearby supermarkets, revealing that only one sample contained 15.6 μg/kg of residual decanoic acid [91]. In another study, Wang et al. developed a rapid and sensitive solvent extraction method for the determination of spectinomycin and lincomycin residues in chicken samples. After derivatization of the target compounds, GC-MS/MS detection was utilized. This method achieved a recovery rate of 80.0% to 95.7%, a relative standard deviation of 1.0% to 3.4%, a detection limit of 2.3 to 4.3 μg/kg, and a quantification limit of 5.6 to 9.5 μg/kg [92]. Guo et al. successfully developed a rapid and sensitive solvent extraction coupled with the GC-MS/MS method for the determination of spectinomycin and lincomycin residues in chicken samples. Following derivatization of the target compounds, the method demonstrated recovery rates of 80.0–95.7%, RSDs of 1.0–3.4%, with LODs ranging from 2.3 to 4.3 μg/kg and LOQs between 5.6 and 9.5 μg/kg [93]. Yu et al. established a GC-MS method to detect and identify amitraz and its metabolite, 2,4-dimethylaniline, in liver and kidney tissues of food-producing animals. At spiked concentrations of 50–300 μg/kg, the method achieved recovery rates of 72.4–101.3%, with RSDs below 11.5% [94].

GC-MS offers several advantages that make it an indispensable tool in analytical chemistry. For example, GC-MS excels at separating and identifying complex mixtures, making it ideal for analyzing pharmaceutical residues in biological matrices [8,95]. GC-MS provides high specificity due to the precise molecular recognition during the ionization process and it is applicable to a wide range of analytes, including organic pollutants, heavy metals, and pharmaceuticals with higher sensitivity [89].

In short, GC-MS is a powerful analytical tool for detecting and quantifying trace volatile organic compounds in complex matrices. Its ability to provide both separation and identification makes it indispensable in food safety monitoring and pharmaceutical residue analysis. However, its application requires careful optimization of the sample preparation and instrumental settings to ensure accurate and reliable results. Despite its limitations, advancements in analytical chemistry continue to enhance its utility and reduce its dependency on expensive equipment, making it more accessible for routine use.

### 3.2. Liquid Chromatography Quadrupole-Time-of-Flight Mass Spectrometry (LC-QTOF-MS)

**Figure 1:**
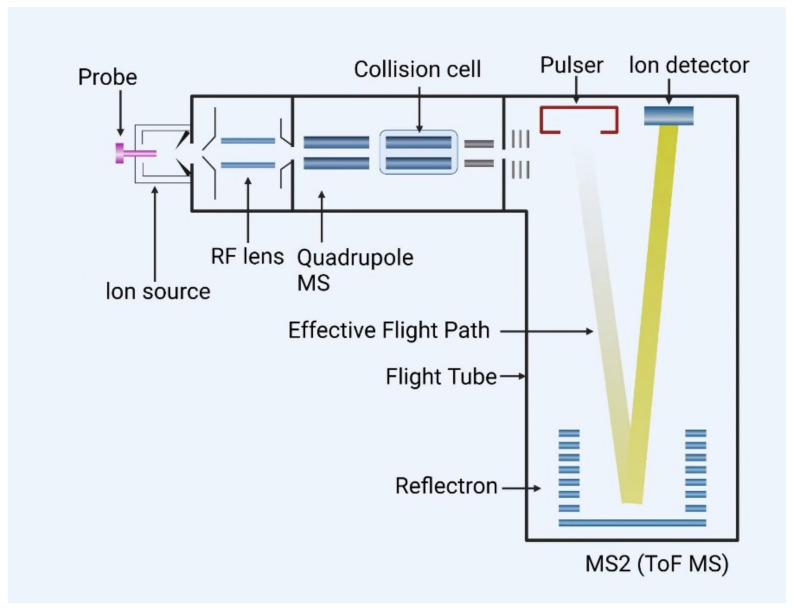
Schematic illustration of the principle of LC-QTOF-MS.

The core principle of LC-QTOF-MS lies in its seamless integration of the separation capabilities of liquid chromatography (LC) with high-resolution, accurate mass measurement of quadrupole time-of-flight mass spectrometry (QTOF-MS) [96]. After sample injection into the liquid chromatography system, different veterinary drug components are separated based on their distribution coefficients between the stationary and mobile phases, as well as their polarity and molecular weight characteristics, as they are eluted sequentially from the chromatographic column driven by the mobile phase. Subsequently, the eluted components enter the QTOF-MS, where they are ionized under the influence of the ion source. The resulting ion beams undergo preliminary screening in the quadrupole to eliminate some interfering ions before entering the time-of-flight analyzer. In the flight tube, ions travel at different speeds depending on their mass-to-charge ratio (m/z), with the flight time inversely proportional to the m/z ratio. The detector accurately records the ion flight times and converts them into mass spectral data, enabling precise determination of ion mass with ultra-high resolution. This generates highly accurate mass spectra, which, through database comparison or analysis of characteristic fragment ions, allow for the precise identification and quantification of trace veterinary drug residues [97,98]. A simple working mode diagram is shown in Figure 1.

LC-QTOF-MS is recognized as a high-resolution mass spectrometer capable of achieving fine resolution for compounds with similar mass-to-charge ratios, making it particularly advantageous for differentiating and characterizing similar ions [99,100]. For instance, Li et al. developed a comprehensive screening method using HPLC-QTOF-MS for the simultaneous determination of 141 veterinary drugs and their metabolites in pork. Most of the tested compounds exhibited a detection limit of 0.5 μg/kg, with recovery rates exceeding 70% [101]. Similarly, Vardali et al. established a UPLC-QTOF-MS method for the concurrent determination of 20 veterinary drug residues and metabolites (including tetracyclines, quinolones, sulfonamides, and diaminopyrimidines) in the edible muscle and skin tissues of European sea bass. The recovery rates for all target compounds in both muscle and skin tissues ranged from 93.8% to 107.5%, with detection and quantification limits spanning 2.22 to 15.00 μg/kg and 6.67 to 45.46 μg/kg, respectively [102].

This technology boasts several notable advantages [95]. Firstly, it excels in the deep separation and precise recognition of veterinary drugs within complex biological matrices. This capability effectively mitigates interference from structurally similar substances, thereby providing reliable and accurate results. Secondly, the technology exhibits exceptional sensitivity. It can detect extremely low concentrations of veterinary drug residues, ensuring compliance with stringent food safety standards and offering a high level of assurance in the detection process. Thirdly, it possesses robust qualitative capabilities. The acquisition of high-resolution mass spectral data yields rich structural information, which aids in the identification of unknown substances [103]. This broadens the scope of detection and enhances the overall analytical power of the technology [104].

However, it is important to acknowledge certain limitations, particularly in terms of cost and data processing. For instance, the acquisition and maintenance of the required instrumentation entail significant expenses. Additionally, substantial investment in laboratory infrastructure and the employment of highly skilled personnel are necessary to operate and maintain the technology effectively [105]. Moreover, the vast amount of high-resolution data generated necessitates the use of specialized software for interpretation. This process can be both time-consuming and labor-intensive. Furthermore, improper handling of the data may introduce errors, which could compromise the accuracy of the detection results [106]. Therefore, meticulous attention to detail and rigorous quality control measures are essential to ensure the reliability and precision of the technology.

In summary, LC-QTOF-MS represents a powerful analytical tool in the field of veterinary drug residue analysis, offering unparalleled precision and sensitivity. Nevertheless, its application demands careful consideration of the associated costs and technical challenges.

### 3.3. Liquid Chromatography-Tandem Mass Spectrometry (LC-MS/MS)

LC-MS/MS is widely used in the field of veterinary drug residue detection [107]. Its working principle is as follows [46]: First, the sample is injected into the liquid chromatography system. Utilizing the differences in distribution coefficients between the mobile phase and the stationary phase, based on properties such as polarity and molecular weight, the various components are eluted from the chromatography column in a specific order, achieving initial separation. Subsequently, the eluted components enter the mass spectrometer, where the molecules are ionized to form ion beams under the action of the ion source. The ions first undergo primary mass spectrometry analysis, where they are separated and screened based on differences in the mass-to-charge ratio (m/z). After screening out the target ions, these ions further collide with an inert gas in the collision chamber, breaking apart to produce characteristic fragment ions, which are then analyzed by secondary mass spectrometry. By comprehensively analyzing the mass-to-charge ratio and abundance information of the parent ions from the primary mass spectrometry and the fragment ions from the secondary mass spectrometry, and comparing them with known databases, precise qualitative and quantitative determination of veterinary drugs is achieved [108].

In recent years, as LC-MS/MS has been developing, it has turned into a commonly utilized method for analyzing trace organic compounds. This technology can identify trace organic compounds at a concentration of 1 μg/kg [109,110]. This technology offers the following significant advantages: It boasts extremely high sensitivity, is capable of detecting trace or even ultra-trace levels of veterinary drug residues, meets increasingly stringent food safety standards, and provides strong support to ensure the quality and safety of animal-derived food products. It exhibits excellent selectivity; through the dual selection and analysis of parent ions and fragment ions, it effectively eliminates many interfering components in complex sample matrices, accurately identifies target veterinary drugs, and reduces the likelihood of false-positive results. Its qualitative and quantitative accuracy is strong, with rich mass spectrometry information combined with professional software algorithms ensuring reliable detection results, making it suitable for the simultaneous detection of multiple veterinary drug residues in complex biological samples [111]. For instance, Lehotay et al. carried out a study regarding the regulatory analysis of veterinary drug residues in liquid and powdered eggs by means of UPLC-MS/MS and evaluated the validation of 169 veterinary drugs in those eggs. When it comes to the extraction and injection sample preparation method, for 139–141 (82–83%) out of 169 different drug analytes added to powder and liquid eggs at three supervisory levels, it achieved acceptable recovery rates ranging from 70 to 120% and a relative standard deviation of less than 25% [112]. Additionally, Na et al. adopted improved QuEChERS and UPLC-MS/MS to confirm the methods for 197 pesticides, 56 veterinary drug components, and 5 toxin components. In the improved analysis method, all components demonstrated good linearity (r2 ≥ 0.98). For most compounds, the limit of quantification is 0.05 mg/kg. And the recovery rate lies between 70.09 and 119.76% with a relative standard deviation of less than 18.91% [113]. Wittenberg et al. established an LC-MS/MS method for the determination of 52 veterinary drug residues in milk powder. The LOQs ranged from 0.02 to 82 ng/g. In skimmed milk powder spiked at three concentration levels, 40 out of the 52 target compounds exhibited satisfactory recovery rates (70–120%) and precision (relative standard deviations, RSDs < 20%). Comparable results were also achieved in whole milk powder, whole milk protein, whey protein concentrate, and whey protein isolate [114].

On the contrary, LC-MS/MS technology also has certain limitations. Firstly, the instruments are expensive, with high acquisition costs, and subsequent maintenance and upkeep require skilled technicians and stringent laboratory environments, increasing detection costs and operational difficulty. Secondly, the operation is complex, as sample preparation, instrument parameter optimization, and data processing all require specialized knowledge and extensive experience, making it challenging for beginners to master [115,116]. Despite these limitations, LC-MS/MS remains a vital tool for ensuring food safety and regulatory compliance.

### 3.4. Liquid Chromatography Coupled to Ion Trap Mass Spectrometry (LC-IT-MS)

LC-IT-MS is grounded in the synergistic operation of liquid chromatography and ion trap mass spectrometry [117]. In this technique, the sample is first introduced into the liquid chromatography system, where different veterinary drug components are sequentially separated based on their distinct distribution coefficients between the mobile and stationary phases. This separation is facilitated by differences in polarity, molecular weight, and other physicochemical properties within the chromatographic column. Subsequently, the separated components are eluted into the ion trap mass spectrometer [118].

Within the ion source, the molecules of veterinary drugs are ionized, and these ions are then introduced into the ion trap. The ion trap employs a radiofrequency electric field to capture, store, and manipulate the ions, allowing for the precise selection of ions with specific mass-to-charge ratios (m/z). By adjusting the parameters of the electric field, the ions are induced to collide with a buffer gas, leading to fragmentation and the generation of multiple-stage fragment ions [117]. Through in-depth analysis of these multi-stage mass spectra, coupled with the use of specialized software and reference databases, accurate identification and quantitative determination of veterinary drugs are achieved [117].

LC-IT-MS addresses the limitations of LC-MS/MS in terms of compound retrieval breadth and is capable of simultaneously confirming and screening multiple trace compounds [119,120,121]. For instance, Kang et al. developed a multi-residue method using ion trap LC-IT-MS to screen for 110 veterinary drug residues. Among these, 108 (98%) were detected at concentrations below 10 ppm. The method demonstrated an average accuracy ranging from 63% to 122% in measuring drug residues in muscle, liver, and kidney tissues, with repeatability and reproducibility ranging from 5% to 22% and 7% to 23%, respectively [122]. Similarly, Casado et al. employed Ultra-Performance Liquid Chromatography-Ion Trap Mass Spectrometry (UPLC-IT-MS) for the simultaneous determination of 23 veterinary drug residues, including beta-blockers, beta-agonists, and non-steroidal anti-inflammatory drugs, in meat samples. The detection limits of this method ranged from 0.01 to 18.75 μg/kg, while the quantification limits spanned from 0.02 to 62.50 μg/kg [123]. Furthermore, analysis of actual beef samples revealed traces of propranolol, ketoprofen, and diclofenac in some instances.

The technique exhibits several notable advantages: (1) It can provide exceptional qualitative capability. The rich fragment ion information provided by multi-stage mass spectrometry effectively distinguishes structurally similar veterinary drugs, significantly enhancing qualitative accuracy and supporting complex sample analysis. (2) LC-IT-MS is highly sensitive to trace levels of veterinary drug residues, meeting stringent food safety standards and ensuring the quality and safety of animal-derived products. (3) Compared to high-end hyphenated instruments, the ion trap mass spectrometer is compact and imposes less stringent requirements on laboratory infrastructure and funding, facilitating widespread adoption [124]. Despite its advantages, LC-IT-MS has certain limitations. (1) The complex motion of ions within the ion trap and uncertainties during collision processes can lead to variations in quantitative results, potentially affecting detection accuracy. (2) The method’s scanning speed is comparatively slow, making it less efficient for rapid data acquisition in multi-component analyses or high-throughput screening scenarios, thereby extending the overall detection cycle and somewhat limiting its application in large-scale, high-throughput detection contexts [125].

In conclusion, LC-IT-MS represents a powerful analytical tool with significant advantages in qualitative analysis and sensitivity, though its limitations in precision and scanning speed warrant consideration in specific applications [126].

### 3.5. CE-MS

The fundamental principle of CE-MS involves the following sequential processes: Initially, a sample solution containing veterinary drug residues is introduced into the capillary electrophoresis system. Under the influence of a high-voltage electric field, the various components of veterinary drugs are separated within the capillary based on differences in their charge, size, and shape. Components with higher electrophoretic mobility reach the end of the capillary first. Subsequently, the separated components enter the mass spectrometer immediately, where they are ionized by the ion source, converting them into ions with specific mass-to-charge ratios (m/z). These ions then move through the mass analyzer according to their m/z values under the influence of magnetic or electric fields, ultimately being detected by the detector to generate a mass spectrum. By comparing the obtained spectra with known standard libraries and analyzing characteristic ion peaks, precise qualitative and quantitative detection of veterinary drugs is achieved [127,128].

The development of CE-MS has addressed the sensitivity limitations traditionally associated with CE. CE is characterized by high separation efficiency, rapid analysis speed, low reagent consumption, and straightforward operation. In the context of detecting residues in animal-based foods, CE-MS is particularly suitable for analyzing both weakly and strongly ionized metabolites, as well as their three-dimensional structures [129,130,131]. For instance, Blasco et al. successfully utilized CE-MS/MS to simultaneously detect eight residual veterinary drugs (ciprofloxacin, enrofloxacin, sulfacetamide, sulfabenzamide, sulfachloropyridazine, sulfaquinoxaline, sulfathiazole, and sulfamethoxazole) in milk. Acetonitrile was employed as the extraction solvent, and the Oasis HLB solid-phase extraction (SPE) column was used for purification. All eight veterinary drugs exhibited recovery rates exceeding 78%. The detection limits of this method ranged from 1 to 9 μg/kg, indicating the feasibility of CE-MS/MS for the initial analysis of veterinary drugs in milk [132].

This technique offers several notable advantages in separation efficiency, sensitivity, and sample requirement [133]. (1) The capillary electrophoresis allows for the fine separation of veterinary drugs based on their molecular properties in a short period, effectively addressing the coexistence of multiple components in complex sample matrices. This reduces peak broadening and enhances resolution. (2) CE-MS can detect trace amounts of veterinary drug residues, meeting increasingly stringent food safety standards and providing robust protection for the quality and safety of animal-derived foods. (3) This technique requires only a small amount of sample, significantly reducing detection costs. This is particularly advantageous for precious samples or situations with limited sample availability. However, CE-MS has several limitations [134]; for instance, in the required instrumentation, the interfacing technology between capillary electrophoresis and mass spectrometry is challenging, requiring precise calibration to ensure their synergistic operation. This places high demands on the technical expertise of the operator. Additionally, in methodological instability, the electrophoretic process is susceptible to variations in temperature and buffer composition, leading to poor reproducibility of migration times and peak areas. Frequent calibration and optimization are necessary, which increases the complexity and uncertainty of the detection process.

## 4. Rapid Detection Techniques

In recent years, a novel detection concept named rapid detection has been put forward. It represents a technical means that can generate detection outcomes within a brief time frame, and this also encompasses the preparation of test samples. When contrasted with traditional detection techniques like common physical–chemical testing and large-scale instrument testing, the rapid detection technologies such as Immunoassay analysis techniques, fluorescence polarization immunoassay, and surface-enhanced Raman scattering, showed superiority in terms of short detection time, uncomplicated instrument equipment, and convenience for on-site online detection [135,136,137].

### 4.1. Immunoassay Analysis Techniques

Immunological methods of veterinary drug residue analysis are based on the highly specific reaction of the substance with antibodies. Currently, various immune-based screening assays for antibiotic residue detection have been developed, such as GICA, ELISA, chemiluminescence immunoassay (CLIA), radioimmunoassay (RIA), and fluorescence immunoassay (FIA), and others [138]. The immunoassays are characterized by their high specificity and sensitivity. In general, the LODs achieved are lower and the analysis time is significantly shorter than that required for some of the microbiological tests. Based on the manipulation of assay formats and the use of broad-specificity antibodies, the multi-analysis of several targets in one single test is available [139].

#### 4.1.1. GICA

The GICA method is a qualitative and semi-quantitative analysis approach that employs colloidal gold as a tracer. It is intercepted at the sites where antigens and antibodies specifically react and then shows color. This method is characterized by high-speed operation and great accuracy [140]. Ziyang et al. created a rapid analysis method which combines ultrasound-assisted SPE and colloidal gold test paper and utilized it to detect the residues of veterinary drugs like chloramphenicol, malachite green, cryptomalachite green, and semicarbazide in common aquatic products. Under the optimized conditions, the detection limit of this method is between 0.01 and 0.5 μg/kg, the absolute recovery rate is within 84.2–112.9%, and the average detection time is 15 min [141]. Na et al. produced a specific mouse monoclonal antibody against nitriles and developed a colloidal gold immunochromatographic test strip based on mouse monoclonal antibodies for screening nitriles in milk. The minimum detection limit was 1.146 ng/mL, and the average detection time was 10 min [142]. GICA possesses the following significant advantages: it is extremely simple to operate, requires no specialized instruments, and the results can be interpreted visually based on color development. This makes it suitable for on-site rapid testing in settings such as farms, slaughterhouses, and farmers’ markets, significantly improving detection efficiency [143]. The assay is fast, typically completed within 5 to 15 min, allowing for timely feedback on veterinary drug residues and facilitating quick decision-making. Additionally, it is cost-effective, with minimal consumables and relatively simple test strip preparation, reducing overall detection costs and enabling large-scale application [144]. However, the sensitivity of GICA is relatively limited, making it less capable of detecting trace amounts of veterinary drug residues, which may not meet the increasingly stringent requirements for low residue limits in food safety standards. Additionally, the qualitative accuracy is suboptimal, as it is susceptible to interference from sample matrices, such as fats and proteins in animal tissues, which can lead to false-positive or false-negative results [145]. Therefore, confirmatory testing using other methods is often necessary to ensure result reliability. Furthermore, quantitative analysis is challenging, as the color intensity of the test strips only provides a rough estimate of the veterinary drug concentration range, making it less suitable for scenarios requiring precise quantification [142,146].

#### 4.1.2. ELISA

**Figure 2:**
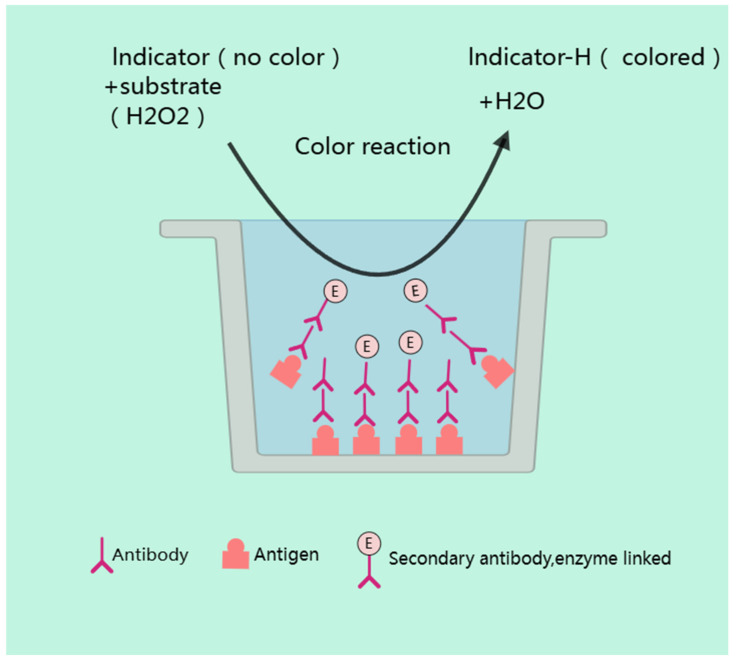
Schematic illustration of the principle of ELISA.

ELISA is a widely used analytical technique in veterinary residue [147]. Its principle is grounded in the specific immune reaction between antigens and antibodies. In this method, a known antigen or antibody is immobilized onto the surface of a solid-phase carrier, such as a microplate. When the tested sample is added, if it contains the target veterinary drug (antigen), it will specifically bind to the immobilized antibody, forming an antigen–antibody complex. Following this initial binding, unbound substances are washed away to ensure specificity. Subsequently, an enzyme-labeled secondary antibody is introduced. This secondary antibody is designed to specifically recognize and bind to the antigen that has already been captured by the primary antibody. After this step, another washing process is performed to remove any unbound enzyme-labeled antibodies. The final stage involves the addition of an enzyme substrate. The enzyme catalyzes a chemical reaction with the substrate, leading to the generation of a colored product [148]. The intensity of this color is directly proportional to the amount of antigen present in the sample. The absorbance of this colored product is then measured using a microplate reader. By comparing the absorbance values to a standard curve, the concentration of the veterinary drug in the sample can be accurately determined [149]. A schematic illustration of the principle is shown in Figure 2.

The ELISA method can produce a color change in enzyme reaction substrates via the specific binding of antigens and antibodies [150]. Fu et al. developed an indirect competitive enzyme-linked immunosorbent assay based on specific monoclonal antibodies for quickly screening estradiol in animal-derived foods. The detection limit and quantification limit are 68.1–103.1 ng/L and 100.8–12.7 ng/L, respectively. The recovery rate of estradiol varies from 70.1% to 103.1%, with a coefficient of variation of less than 13.9% [151]. Yin et al. prepared a monoclonal antibody with high specificity and sensitivity and developed an indirect competitive enzyme-linked immunosorbent assay to detect praziquantel. The limit of detection calculated from the analysis of broccoli, cabbage, wheat, corn, rice, chicken, fish, and crayfish samples was 1.56–2.72 mug/kg, and the average recovery rate was 81.25–103.19% [152]. The ELISA method usually has several advantages. In the sensitivity, it can detect trace amounts of veterinary drug residues, following the stringent food safety standards and provide strong support for ensuring the quality and safety of animal-derived foods. Additionally, its specificity is strong, as the precise binding of antigens and antibodies ensures highly selective recognition of the target veterinary drug, effectively excluding interference from other impurities in the sample and ensuring accurate results. Moreover, its operation is relatively simple, requiring no complex instruments; conventional laboratory equipment such as microplates and pipettes can be used. Multiple samples can be processed simultaneously, making it suitable for batch testing and significantly improving detection efficiency [153,154]. However, the ELISA method also has some limitations. There is a risk of cross-reactivity, where antibodies may misidentify structurally similar veterinary drugs or their metabolites, leading to false-positive results and affecting detection accuracy. Enzyme activity is susceptible to environmental factors such as temperature and pH, requiring strict control of operating conditions. Improper control can reduce enzyme activity, thereby affecting the reliability of the results [155].

### 4.2. Fluorescence Polarization Immunoassay (FPIA)

**Figure 3:**
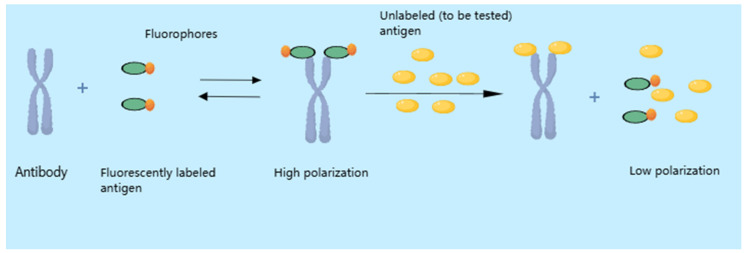
Schematic illustration of the principle of FPIA.

FPIA exhibits distinct advantages in veterinary drug residue detection, leveraging the principle of fluorescence polarization changes induced by specific antigen–antibody binding reactions [156]. In this method, a fluorophore-labeled veterinary drug antigen is introduced into a reaction system containing specific antibodies along with the sample. In the solution state, if the sample is devoid of veterinary drug residues, the fluorophore-labeled antigen can fully bind with the antibodies, forming large molecular complexes. This results in a reduction in rotational speed, thereby increasing the degree of fluorescence polarization. Conversely, if the sample contains the target veterinary drug, due to the competitive binding mechanism, it will preferentially bind with the antibodies, reducing the chance for the fluorophore-labeled antigen to bind with the antibodies [157]. Consequently, fewer complexes are formed, and the number of fluorophore-labeled antigens in small molecular states increases, leading to faster rotational speeds and a decrease in fluorescence polarization. By utilizing a fluorescence polarization analyzer to measure the fluorescence polarization value and comparing it with a standard curve, the amount of veterinary drug residue can be determined [158,159]. A schematic illustration of the principle of FPIA is shown in Figure 3.

FPIA usually provides rapid detection, typically completing the measurement of a single sample within minutes, making it suitable for on-site rapid screening. The technique exhibits considerable sensitivity, capable of detecting trace amounts of veterinary drugs, which is crucial for ensuring food safety and controlling the misuse of veterinary drugs, aligning with current stringent regulatory limits [160]. Additionally, FPIA is straightforward to operate, requiring no complex sample pretreatment steps. The process involves simple mixing and measurement, and it does not demand advanced technical skills from operators, making it user-friendly [161]. For example, Duan et al. created a rapid FPIA for the measurement of erythromycin in milk. To attain high sensitivity, five erythromycin tracers having diverse fluorescent structures were synthesized and paired with three monoclonal antibodies for detecting erythromycin in milk. The detection limit reached 14.08 μg/L and the recovery rate ranged from 96.08 to 107.77%. The overall testing time from adding the sample to obtaining the result reading was less than 5 min [162]. Duan et al. also devised a uniform high-throughput screening dual-wavelength FPIA for determining sulfamethoxazole and trimethoprim in milk, with recovery rates of 81.7–97.2% and 78.6–103.6%, respectively, and a coefficient of variation under 18.9%. Including sample pretreatment, it could be accomplished within 15 min [163].

FPIA has notable limitations. The instrumentation, such as fluorometers with polarization modules, involves significant upfront costs, which can deter small-scale laboratories. Its detection scope is restricted by the availability of high-affinity antibodies and fluorescent tracers, particularly for veterinary drugs with complex structures or low immunogenicity, limiting multi-residue analysis [164]. Additionally, matrix components (e.g., fats or proteins in animal tissues) can interfere with fluorescence polarization signals, necessitating pre-treatment steps like solid-phase extraction or dilution to ensure accuracy [165]. While FPIA offers rapid, homogeneous detection, these challenges highlight the need for complementary methods in complex residue analysis.

### 4.3. Surface-Enhanced Raman Scattering (SERS)

**Figure 4:**
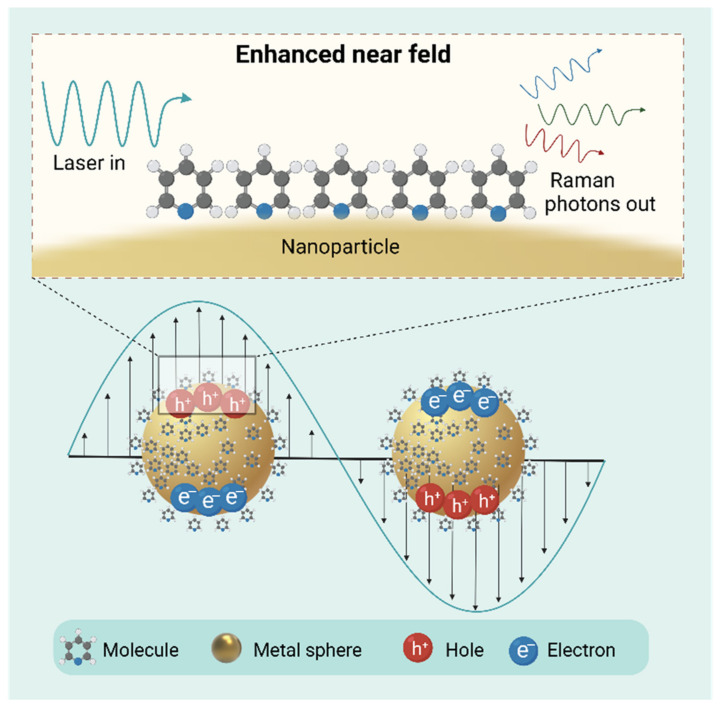
Schematic illustration of the principle of SERS.

SERS is founded on the Raman scattering principle (shown in Figure 4). When pyridine molecules are adsorbed on rough silver electrodes, the Raman scattering signal can be enhanced by 4–6 orders of magnitude. Through the combination of SERS technology and various materials, the detection of small molecules in veterinary drug residues becomes possible [166,167,168]. Dhakal et al. worked out a straightforward surface-enhanced Raman spectroscopy approach for on-the-spot screening of tetracycline residues in milk and water. In water–tetracycline solutions, tetracycline vibration modes were spotted at 1285, 1317, and 1632 cm^−1^, while in milk–tetracycline solutions, they were seen at 1322 and 1621 cm^−1^. A tetracycline residue concentration as minuscule as 0.01 ppm was detected in both solutions. The peak intensities at 1285 and 1322 cm^−1^ were utilized to calculate the tetracycline concentrations in water and milk, with correlation coefficients of 0.92 for water and 0.88 for milk [169]. Fan et al. devised a lateral flow immunosensor depending on dual SERS for the concurrent detection of tetracycline and penicillin residues in milk. Two nanoprobes are capable of recognizing tetracycline-BSA and ampicillin-BSA, respectively, which makes it easier to detect these two kinds of antibiotics simultaneously on a single test line. The detection limits for tetracycline and penicillin are as low as 0.015 ng/mL and 0.010 ng/mL correspondingly, with recovery rates varying from 88.8% to 111.3% and relative standard deviation less than 16% [170].

SERS technology offers significant advantages such as it exhibits extremely high sensitivity, enabling the detection of trace and even ultra-trace levels of veterinary drug residues. This capability is crucial for meeting stringent food safety standards and ensuring the quality and safety of animal-derived products, as it effectively monitors the use of veterinary drugs at very low concentration levels [171]. The detection process is rapid, with spectral acquisition and analysis typically completed in a short time, making it suitable for on-site rapid screening scenarios such as farmers’ markets and customs quarantine [172]. This allows for the timely identification of problematic products, thereby enhancing regulatory efficiency. Additionally, SERS requires minimal sample pretreatment, as it can directly analyze raw or simply processed samples, reducing errors and time associated with extensive preprocessing and simplifying the detection workflow [173]. Finally, SERS testing requires Raman spectrometers and suitable SERS active substrates, and has low-cost manufacturability and commercial accessibility.

However, SERS is mostly confined to academic laboratories and is not recognized as a gold-standard analytical technique, especially in the field of veterinary drug residue detection. This is due to several certain limitations, including its reproducibility is relatively poor. The Raman enhancement effect is highly susceptible to factors such as the size, shape, and aggregation state of metal nanoparticles, as well as the adsorption mode between the sample and nanoparticles. These variables can lead to significant fluctuations in results across different experimental batches or detection positions, compromising data reliability [174]. Quantitative analysis is challenging; although SERS can qualitatively identify veterinary drugs, the complex enhancement mechanisms, and matrix interferences make it difficult to establish precise quantitative models. The linear relationship between spectral signal intensity and veterinary drug concentration is hard to control accurately, posing challenges for the precise determination of residue levels [175,176].

## 5. Comparative Analysis of Merits and Limitations in Contemporary Veterinary Drug Residue Analytical Techniques for Animal-Derived Food Products

We systematically compared the advantages and disadvantages of the current main methods for detecting veterinary drug residues (as illustrated in Table 2). Of note, GC-MS requires meticulous preparation and optimization, presents certain cost limitations, and is particularly effective for volatile compounds [177]. LC-QTOF-MS offers high-resolution analysis but is constrained by elevated operational costs and complex data processing requirements, making it suitable for complex matrices. LC-MS/MS, while highly sensitive and applicable to numerous food types, is both expensive and technically demanding to operate [177]. LC-IT-MS, despite its slower scan speed, provides a more economical option for preliminary screening purposes. CE-MS achieves high separation efficiency but faces technical implementation challenges, though it is particularly effective for ionized metabolites [178]. Taken together, these established chromatographic platforms, such as GC-MS (LOD 0.1–5 μg/kg for volatile compounds) and LC-MS/MS, demonstrate unparalleled sensitivity and accuracy (85–115% recovery rates), albeit constrained by prolonged analysis cycles, substantial operational costs, and stringent laboratory requirements. In contrast, rapid immunoassays and SERS-based approaches enable on-site screening within 5 min at <10% of instrumental costs, particularly valuable for high-throughput surveillance (200 samples/hour) and resource-limited settings, despite relatively compromised sensitivity and occasional cross-reactivity issues. This technological dichotomy underscores its importance in developing integrated detection paradigms. A hierarchical strategy combining rapid preliminary screening (5–10% presumptive positives) with subsequent instrumental confirmation achieves 300–500% efficiency enhancement while maintaining regulatory-grade reliability. Such synergistic systems address critical industry challenges posed by quick growth in global veterinary drug consumption (for example, global antibiotic sales are expected to increase from 93,309 tons in 2017 to 104,079 tons by 2030) [179], particularly enabling real-time monitoring across decentralized supply chains and rapid response to contamination emergencies. This dual-track approach not only optimizes resource allocation but also establishes an adaptive framework for emerging veterinary pharmacopeia, positioning itself as an indispensable component in modern food safety ecosystems. Future research should prioritize the standardization of validation protocols and the development of multiplexed detection platforms to fully realize the transformative potential of rapid screening technologies in global food surveillance networks.

## 6. Conclusions and Further Prospects

Recent advancements in pre-treatment methods and detection technologies for veterinary drug residues in animal-derived foods have significantly bolstered food safety and regulatory adherence. Innovations in sample preparation, such as SPE, QuEChERS, and MIPs, have streamlined the extraction and purification of complex matrices. These methods have reduced both time and solvent usage while enhancing selectivity [180].

Simultaneously, cutting-edge detection technologies, including LC-IT-MS, LC-MS/MS, biosensors, and nanomaterial-based assays, now facilitate ultrasensitive, multi-residue analysis with high specificity. These technological strides address critical challenges such as low residue concentrations, matrix interference, and the necessity for rapid, high-throughput screening. The integration of automation, artificial intelligence (AI) for data analysis, and portable devices has further revolutionized the field, enabling real-time monitoring and on-site testing. Such progress not only ensures compliance with stringent global regulations (e.g., EU Maximum Residue Limits [MRLs], FDA guidelines) but also mitigates public health risks associated with antibiotic resistance, allergic reactions, and chronic toxicity from veterinary drug residues [181,182].

In resource-limited regions such as China and other developing nations, the development of veterinary drug residue monitoring systems requires strategic methodology selection that prioritizes cost-efficiency, technical accessibility, and large-scale implementation ability. For sample pretreatment, the QuEChERS approach demonstrates superior practicality through its solvent-minimized workflow (<10 mL acetonitrile/sample), equipment independence (requiring only basic centrifugation), and broad matrix compatibility spanning meat, dairy, and aquatic products [183]. Field-deployable detection platforms, particularly GICA and ELISA, achieve reliable on-site screening with rapid turnaround (≤30 min/test) and operator-friendly protocols, maintaining 89–94% concordance with reference methods in multi-laboratory validations [184]. However, advanced techniques like LC-MS/MS provide unparalleled sensitivity and multi-residue capabilities (detection limits 0.1–5 μg/kg, >200 simultaneous analytes), their reliance on expensive instrumentation (about USD500,000+ instrumentation costs) and specialized expertise limits their feasibility in underfunded regions. Therefore, a hybrid strategy combining QuEChERS-based pretreatment with GICA/ELISA screening, supplemented by targeted LC-MS/MS confirmation in centralized facilities, offers a balanced approach to ensure food safety without compromising accessibility [185,186]. This framework aligns with the socioeconomic realities of third-world countries, where cost-efficient, field-deployable solutions are critical for large-scale monitoring and regulatory compliance.

The next-generation paradigm for veterinary drug residue analysis in animal-derived foods necessitates synergistic advancements in pretreatment and detection technologies. In sample preparation, intelligent precision extraction strategies are emerging, such as biomimetic molecularly imprinted nanoreactors that achieve a higher recovery rate through size-exclusion mechanisms, coupled with AI-driven automated platforms capable of processing multiple samples in a short time. Additionally, ultrasensitive detection breakthroughs including CRISPR-Cas12a-mediated cascades and plasmonic metasurface-enhanced Raman spectroscopy utilizing gold nanorod arrays with deep learning deconvolution, can further enhance detection accuracy and speed [187,188,189]. Additionally, developing universal protocols for the simultaneous detection of diverse veterinary drugs (antibiotics, hormones, and anthelmintics) remains crucial, especially with the emergence of novel drug formulations. Harmonizing detection thresholds and methodologies across regions will reduce trade barriers and ensure equitable food safety standards. Enhanced surveillance of veterinary drug residues in food chains is vital to combat antimicrobial resistance, requiring robust collaboration between governments, industries, and researchers. Public education on residue risks and the implementation of transparent labeling systems (e.g., blockchain traceability) can empower consumers to make informed choices and foster trust in food systems. By addressing these areas, the scientific community can continue to advance the safety and quality of animal-derived foods, ensuring they meet the highest standards of public health and regulatory compliance.

## Figures and Tables

**Table 1:** A comprehensive systematic comparison among various pretreatment methods for veterinary drug residues in animal-derived foods.

Method	LOD (μg/kg)	Accuracy (Recovery Rate)	Applicability	Advantages	Disadvantages	Feasibility	Cost-Effectiveness
LLE	0.5–1.0	71.4–120%	Suitable for lipophilic drugs in aquatic products, milk, and tissues.	Excellent selectivity for lipophilic drugs. Simple operation with minimal equipment. Low cost.	High solvent consumption. Prone to emulsification. Limited extraction efficiency for polar analytes.	Simple but requires solvent management.	Low
SPE	0.2–3.0	60–120%	Broad applicability for diverse matrices (honey, muscle, milk, and eggs).	Effective enrichment of trace residues. High selectivity with tailored adsorbents. Reduced solvent use.	High adsorbent costs. Complex optimization for matrix effects. Susceptible to column clogging.	Requires skilled optimization.	Moderate to high
IAC	0.04–0.10	74.5–105%	Ideal for specific targets (e.g., chloramphenicol and β-agonists) in muscle/liver.	Exceptional specificity via antibody–antigen binding. High sensitivity for trace residues. High-throughput potential.	Antibody development is costly/time-consuming. Limited column lifespan. Cross-reactivity risks.	Antibody-dependent and storage-sensitive.	High
QuEChERS	0.15–3.03	52.1–138.2%	Effective for high-fat matrices (beef and chicken) and multi-residue analysis.	Rapid and simple. Cost-effective with minimal solvents. Effective impurity removal.	Sorbent selectivity limitations. Residual matrix interference. Optimization challenges for diverse analytes.	Easy to implement with standard lab tools.	Low to moderate
MIT	0.05–0.5	68.6–95.5%	Customizable for antibiotics (e.g., tetracyclines and β-agonists) in complex matrices.	Tailored specificity via imprinting. Reusable and stable. Adaptable to diverse targets.	Labor-intensive synthesis. Cross-reactivity with structural analogs. Requires confirmatory methods.	Specialized expertise needed for polymer design.	Moderate

**Table 2:** A comprehensive systematic comparison among various detection techniques for veterinary drug residues in animal-derived foods.

Method	LOD (μg/kg)	Accuracy (Recovery Rate)	Applicability	Advantages	Disadvantages	Feasibility	Costing
GC-MS	2.3–4.3	77.38–95.7%	Volatile/semi-volatile compounds.	High specificity for volatile analytes. Robust qualitative capabilities. Wide applicability for small molecules.	Requires derivatization for non-volatile compounds. Limited to thermally stable analytes.	Requires derivatization expertise.	Moderate to high
LC-QTOF-MS	0.5	More than 70%	High-resolution multi-residue screening.	Ultra-high resolution for accurate mass identification. Broad-spectrum detection. Rich structural data.	High equipment/maintenance costs. Demands advanced data analysis skills.	Requires high-end infrastructure.	Very high
LC-MS/MS	0.02–82	70–120%	Gold standard for trace-level quantification.	High sensitivity and selectivity. Reliable for multi-residue analysis. Robust quantitative accuracy.	Expensive instrumentation. Complex sample preparation. Matrix effects require mitigation.	Skilled operation and maintenance needed.	High
LC-IT-MS	0.01–18.75	63–122%	Multi-stage fragmentation for structural elucidation.	Mul-ti-stage mass for structural insights. Compact and cost-effective.	Slower scanning speeds. Moderate resolution limits complex mixture analysis.	Suitable for targeted analysis.	Moderate
CE-MS	1–9	More than 78%	Ionizable metabolites.	High separation efficiency. Minimal sample/reagent consumption. Fast analysis.	Poor reproducibility due to buffer/temperature sensitivity.	Technically demanding for calibration.	Moderate
GICA	0.01–0.5	84.2–112.9%	Rapid on-site screening.	Equipment-free, rapid results. Low cost and user-friendly.	Qualitative/semi-quantitative only. Limited sensitivity for trace residues. Matrix interference risks.	Ideal for field testing.	Low
ELISA	1.56–2.72	70.1–103.1%	High-throughput screening.	High throughput and specificity. Cost-effective for batch analysis. Minimal instrumentation.	Cross-reactivity with analogs. Enzyme activity affected by environmental factors.	Requires antibody development.	Low to moderate
FPIA	0.01	78.6–107.77%	Homogeneous assays.	Rapid and homogeneous. Minimal sample pretreatment. Moderate sensitivity.	Limited by antibody/tracer availability. Matrix interference in complex samples.	Suitable for simple matrices.	Moderate
SERS	0.01–0.015	88.8–111.3%	Ultra-trace detection.	Ultra-high sensitivity. Rapid and minimal pretreatment. Multiplexing potential.	Poor reproducibility due to nanoparticle variability.	Requires nanoparticle optimization.	High

## Data Availability

All data were included in this manuscript.
